# Correction: Zhao et al. Artesunate Inhibits Metastatic Potential in Cisplatin-Resistant Bladder Cancer Cells by Altering Integrins. *Cells* 2025, *14*, 570

**DOI:** 10.3390/cells15151363

**Published:** 2026-07-29

**Authors:** Fuguang Zhao, Olesya Vakhrusheva, Sascha Dennis Markowitsch, Kimberly Sue Slade, Maximilian Peter Brandt, Igor Tsaur, Jindrich Cinatl, Martin Michaelis, Thomas Efferth, Roman Alexander Blaheta, Axel Haferkamp, Eva Juengel

**Affiliations:** 1 Department of Urology and Pediatric Urology, University Medical Center Mainz, Langenbeckstr. 1, 55131 Mainz, Germany; doctor-zfg@outlook.com (F.Z.); olesya.vakhrusheva@med.uni-tuebingen.de (O.V.); sascha.markowitsch@unimedizin-mainz.de (S.D.M.); kimberlysue.slade@unimedizin-mainz.de (K.S.S.); maximilian.brandt@unimedizin-mainz.de (M.P.B.); igor.tsaur@med.uni-tuebingen.de (I.T.); roman.blaheta@unimedizin-mainz.de (R.A.B.); axel.haferkamp@unimedizin-mainz.de (A.H.); 2 Department of Urology, University Hospital Tübingen, Hoppe-Seyler-Strasse 3, 72076 Tübingen, Germany; 3 Interdisciplinary Laboratory for Paediatric Tumour and Virus Research, Dr. Petra Joh Research Institute, 60529 Frankfurt am Main, Germany; j.cinatl@kinderkrebsstiftung-frankfurt.de (J.C.J.); m.michelis@kent.ac.uk (M.M.); 4 School of Natural Sciences, University of Kent, Canterbury CT2 7NJ, UK; 5 Institute of Pharmaceutical and Biomedical Sciences, Johannes Gutenberg University Mainz, Staudinger Weg 5, 55128 Mainz, Germany; efferth@uni-mainz.de

To better reflect the first two authors’ contributions in the original article [1], the authorship order has been revised, and the following back matter statements have been updated:

**Author Contributions**

Conceptualization, E.J.; data curation, F.Z., O.V. and I.T.; formal analysis, F.Z.; funding acquisition, J.C.J., M.M. and E.J.; investigation, E.J.; methodology, F.Z., S.D.M., K.S.S., M.P.B., J.C.J. and M.M.; project administration, E.J.; resources, J.C.J., M.M., T.E. and E.J.; software, F.Z.; supervision, E.J.; validation, O.V.; visualization, F.Z. and O.V.; writing—original draft, F.Z., O.V. and E.J.; writing—review and editing, T.E., R.A.B., A.H. and E.J. R.A.B. is an expert on integrins and therefore had a closer look at the data. He gave several impulses for data presentation and the discussion part. A.H. reviewed the manuscript with his huge expertise regarding urologic cancers and the challenge of treating them in the metastatic stage. He had a great impact on the structure and therewith the end version of the manuscript. All authors have read and agreed to the published version of the manuscript.

**Data Availability Statement**

The raw data supporting the conclusions of this article will be made available by the authors on request. All data are from F.Z.’s Ph.D. thesis, which is archived in the library of the Johannes Gutenberg University Mainz.

These corrections are solely administrative in nature and concern authorship, attribution, and transparency regarding the origin of the data. The updates do not affect the scientific content, methodology, results, interpretation, or conclusions of the article. Accordingly, the validity, integrity, and reliability of the reported findings remain fully unchanged.
