# Supplementary material for: Artesunate Inhibits Metastatic Potential in Cisplatin-Resistant Bladder Cancer Cells by Altering Integrins
Source: Cells. 2025 Apr 10;14(8):570. doi: 10.3390/cells14080570 (PMC12026051; doi:10.3390/cells14080570)
Supplement: Supplementary file 1 [file cells-14-00570-s001.zip › cells-3515190-supplementary.pdf]

Figure1S. Detailed information about Figure 5 – Total protein expression of integrin proteins and integrin-related signaling proteins in both parental and cisplatin-resistant RT112 and T24 cells.

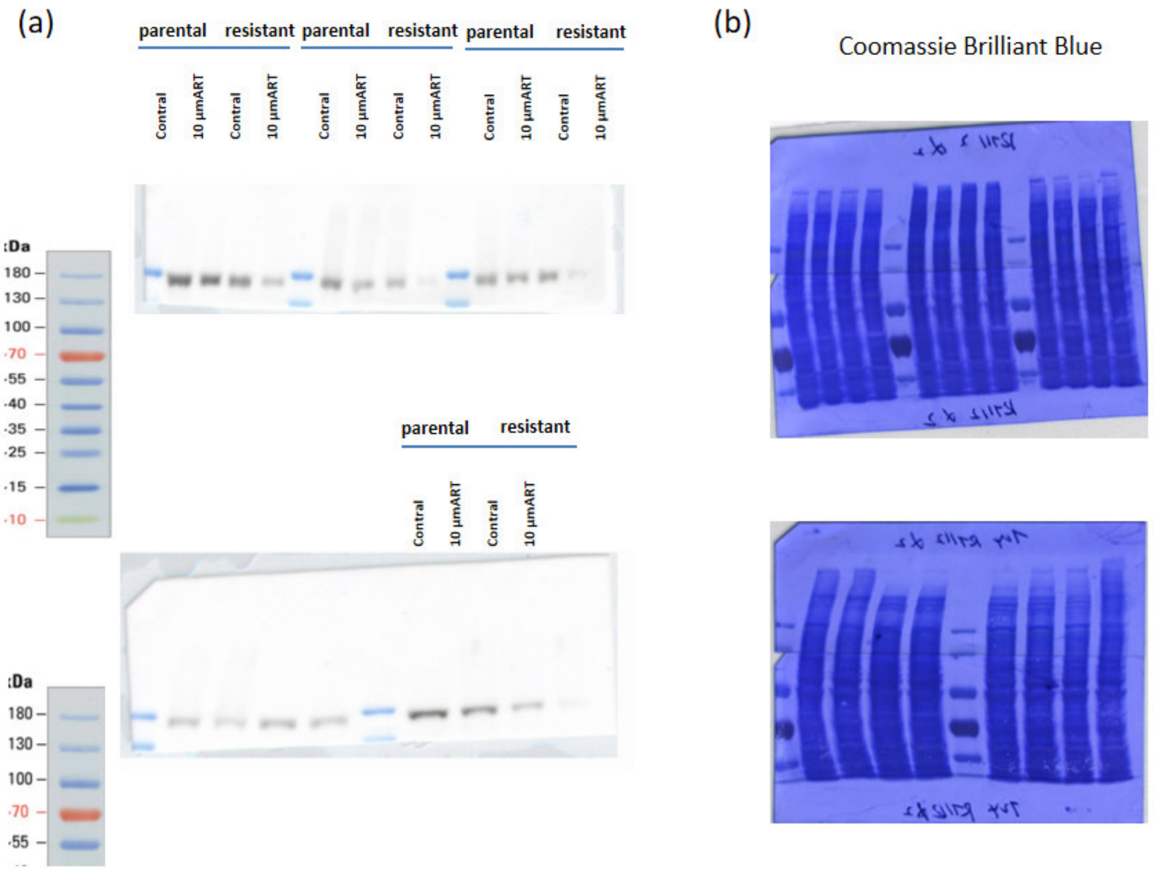

Figure S1.1: Detailed information about Figure 5 - Total protein expression of integrin proteins and integrin-related signaling proteins of RT112 cells. (a) Protein expression of integrin α2 (150 kDa) and (b) corresponding Coomassie blue staining of total protein.

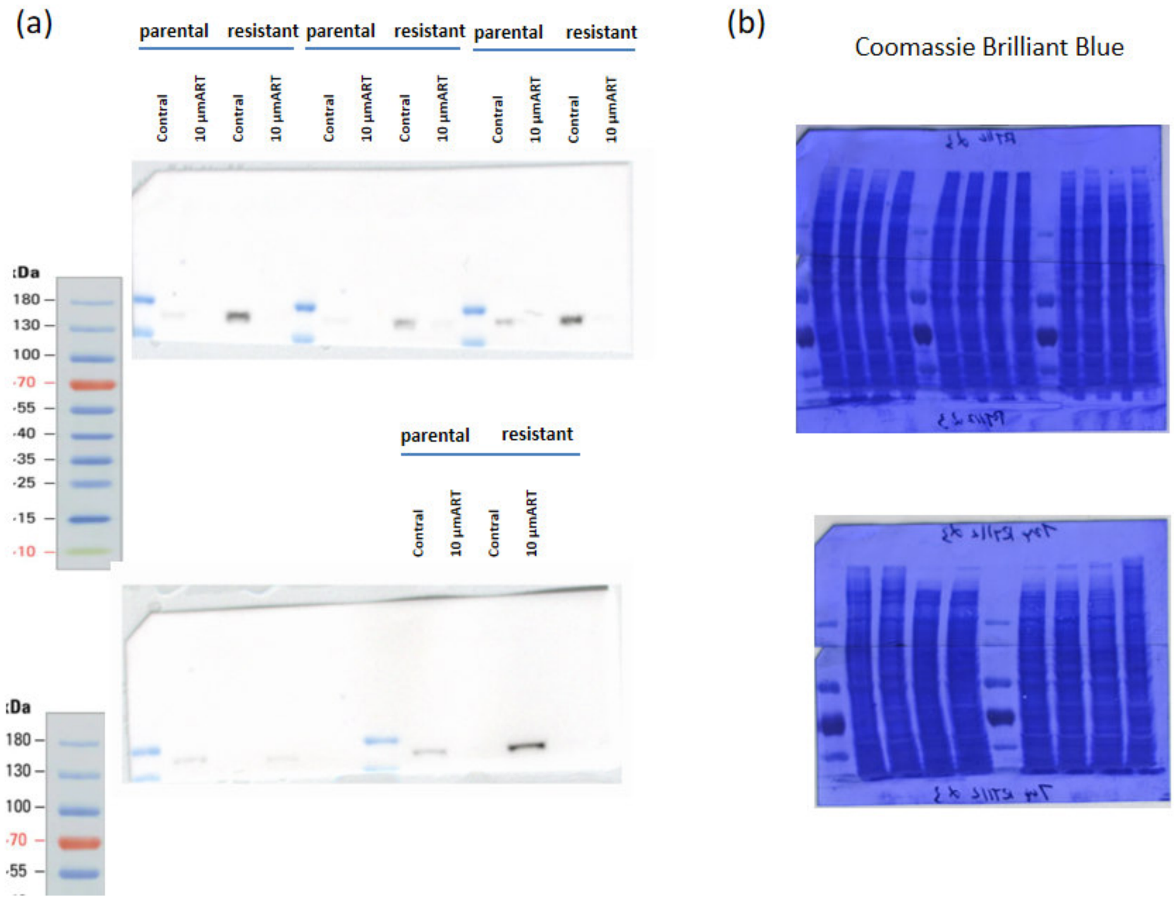

Figure S1.2: Detailed information about Figure 5 - Total protein expression of integrin proteins and integrin-related signaling proteins of RT112 cells. (a) Protein expression of integrin α3 (150 kDa) and (b) corresponding Coomassie blue staining of total protein.

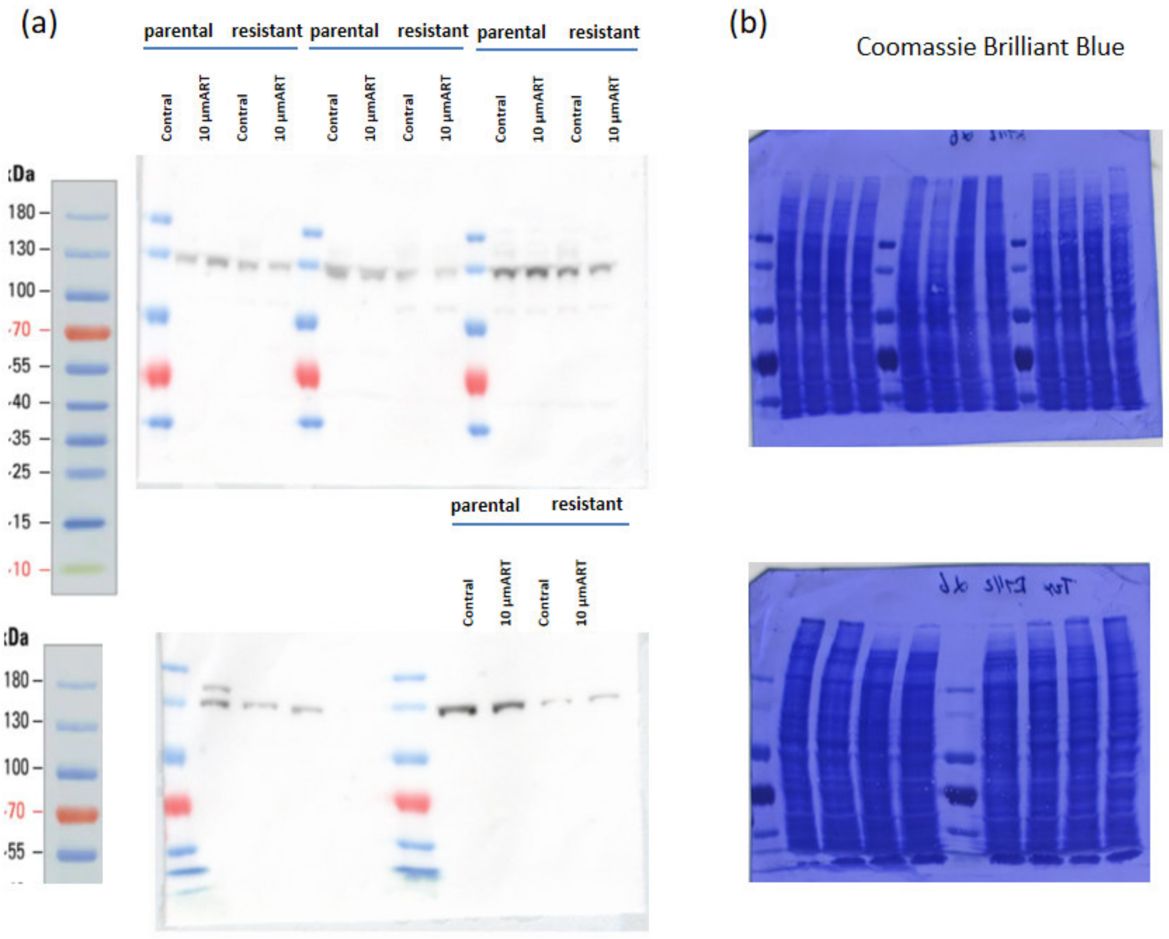

Figure S1.3: Detailed information about Figure 5 - Total protein expression of integrin proteins and integrin-related signaling proteins of RT112 cells. (a) Protein expression of integrin α6 (150,125 KDa) and (b) corresponding Coomassie blue staining of total protein.

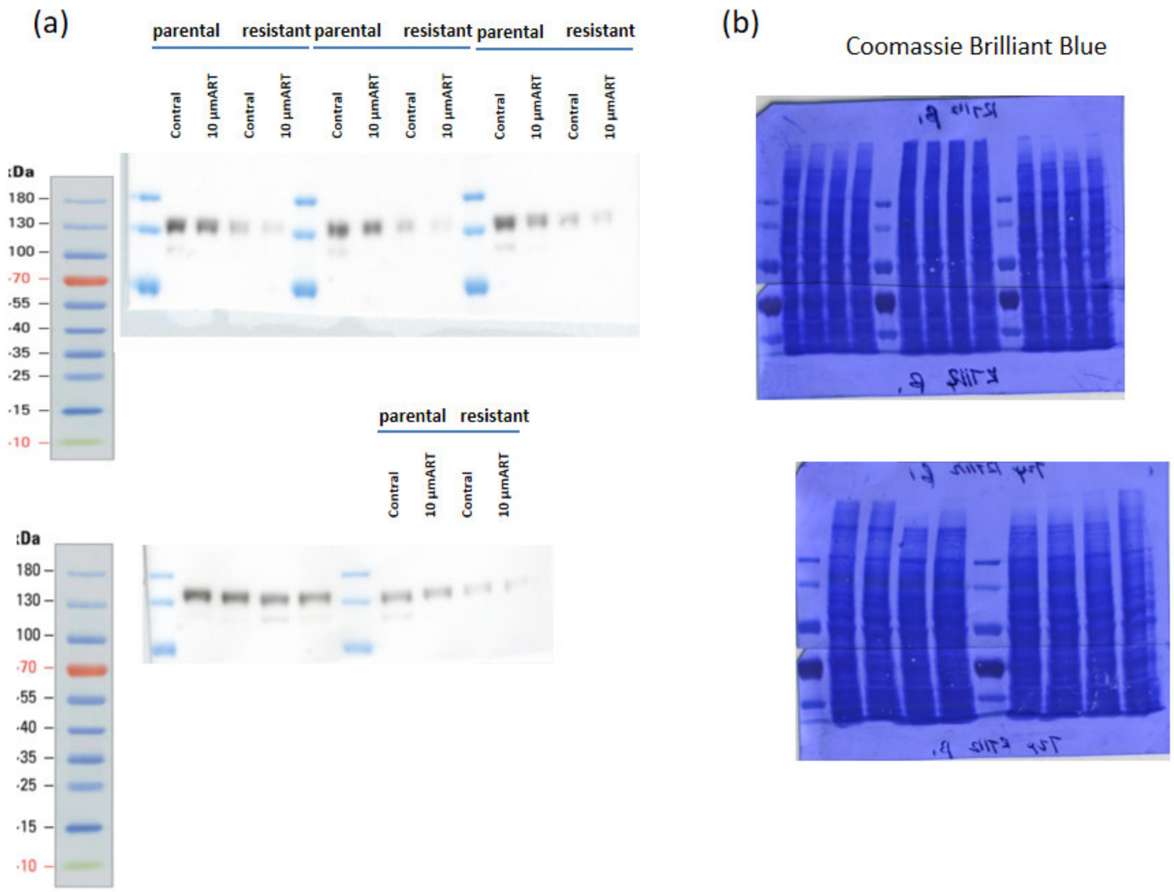

Figure S1.4: Detailed information about Figure 5 - Total protein expression of integrin proteins and integrin-related signaling proteins of RT112 cells. (a) Protein expression of integrin β1 (130 kDa) and (b) corresponding Coomassie blue staining of total protein.

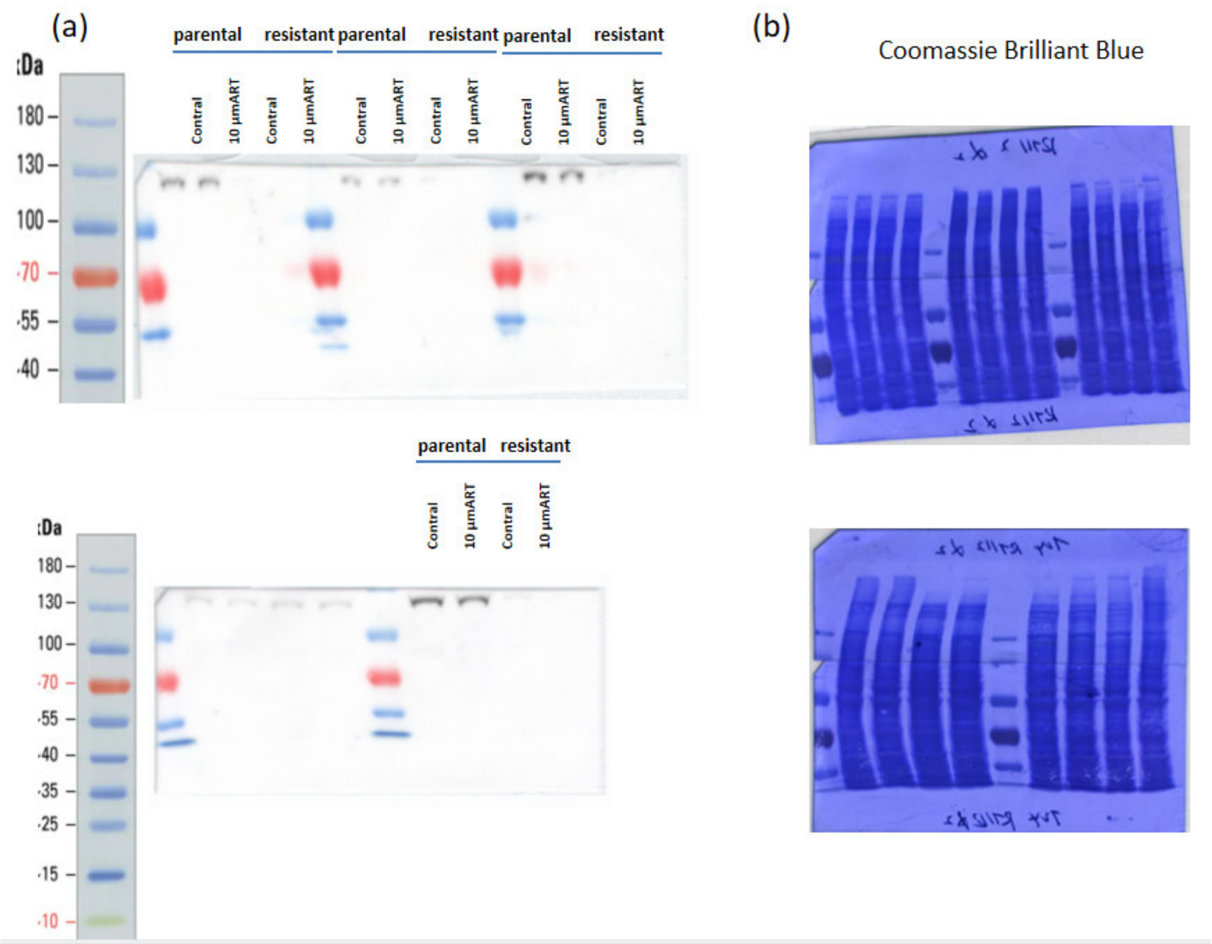

Figure S1.5: Detailed information about Figure 5 - Total protein expression of integrin proteins and integrin-related signaling proteins of RT112 cells. (a) Protein expression of FAK (116-125 kDa) and (b) corresponding Coomassie blue staining of total protein.

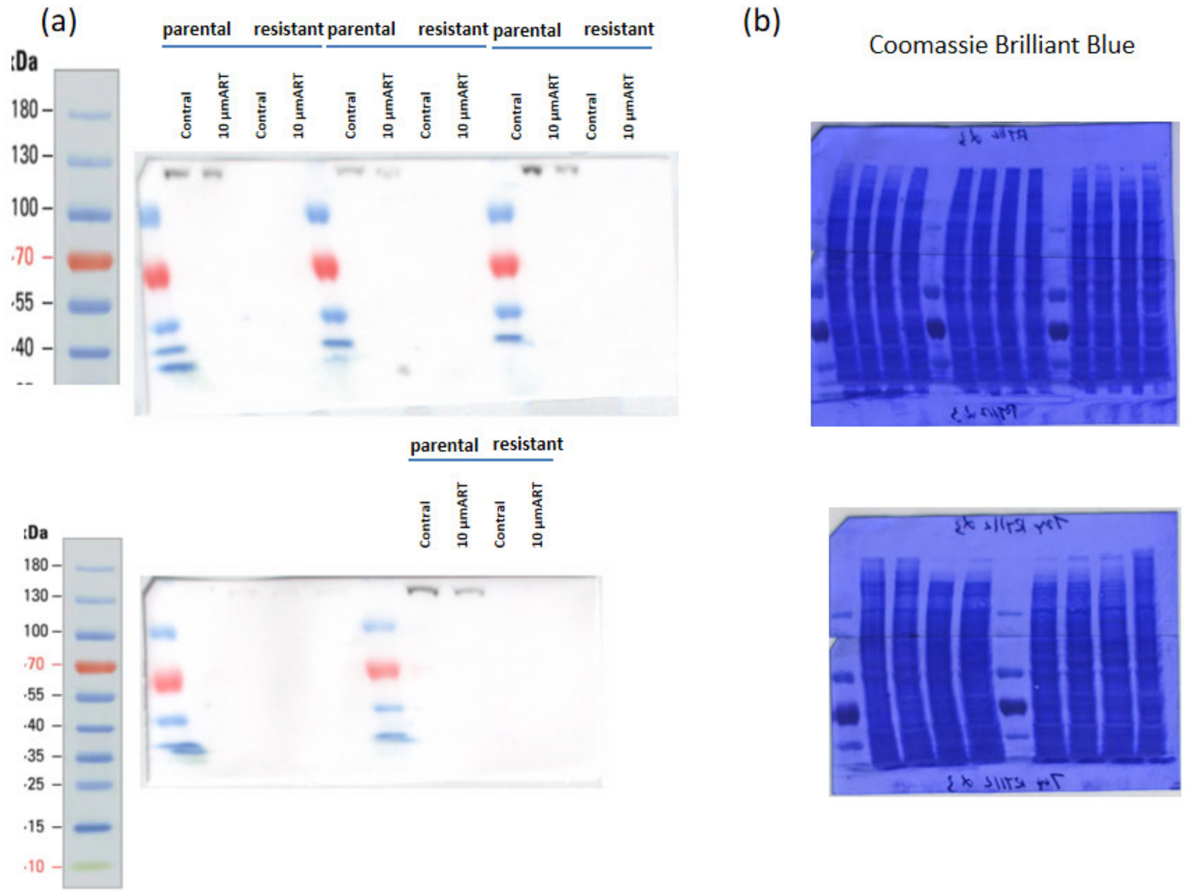

Figure S1.6: Detailed information about Figure 5 - Total protein expression of integrin proteins and integrin-related signaling proteins of RT112 cells. (a) Protein expression of pFAK (116-125 kDa) and (b) corresponding Coomassie blue staining of total protein.

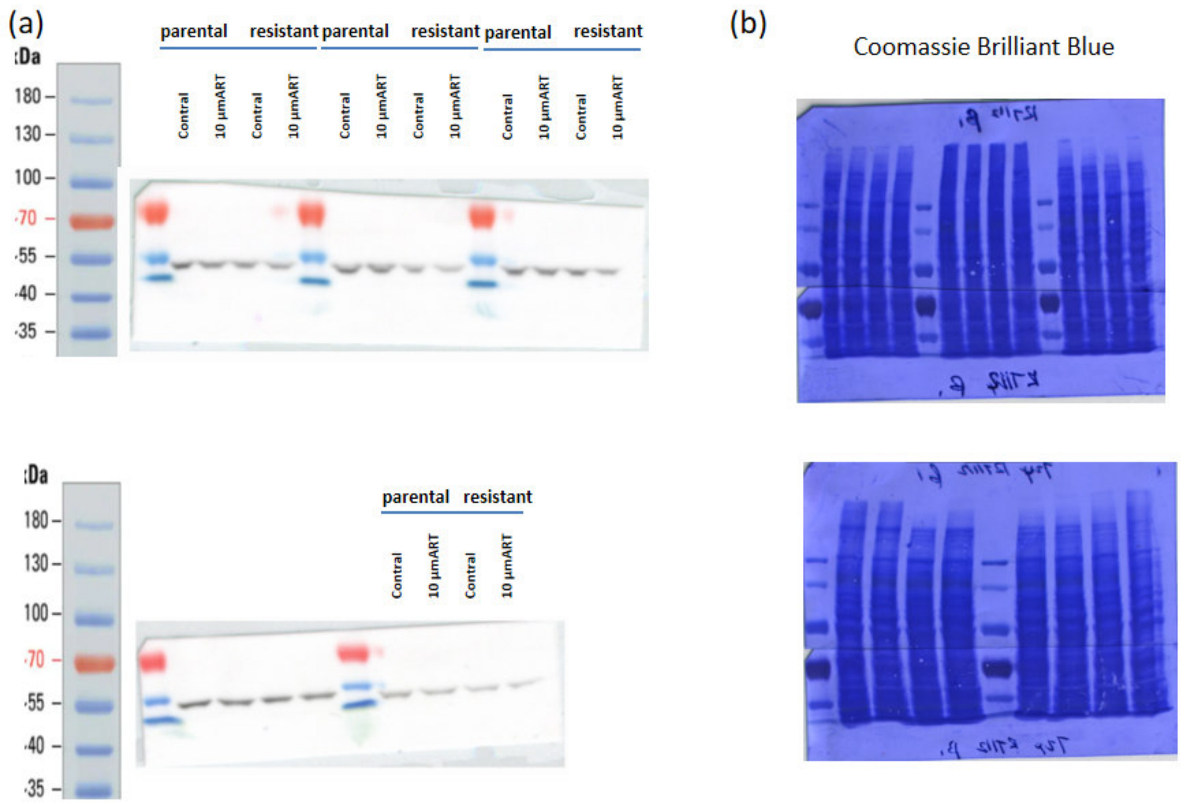

Figure S1.7: Detailed information about Figure 5 - Total protein expression of integrin proteins and integrin-related signaling proteins of RT112 cells. (a) Protein expression of ILK (50 kDa) and (b) corresponding Coomassie blue staining of total protein.

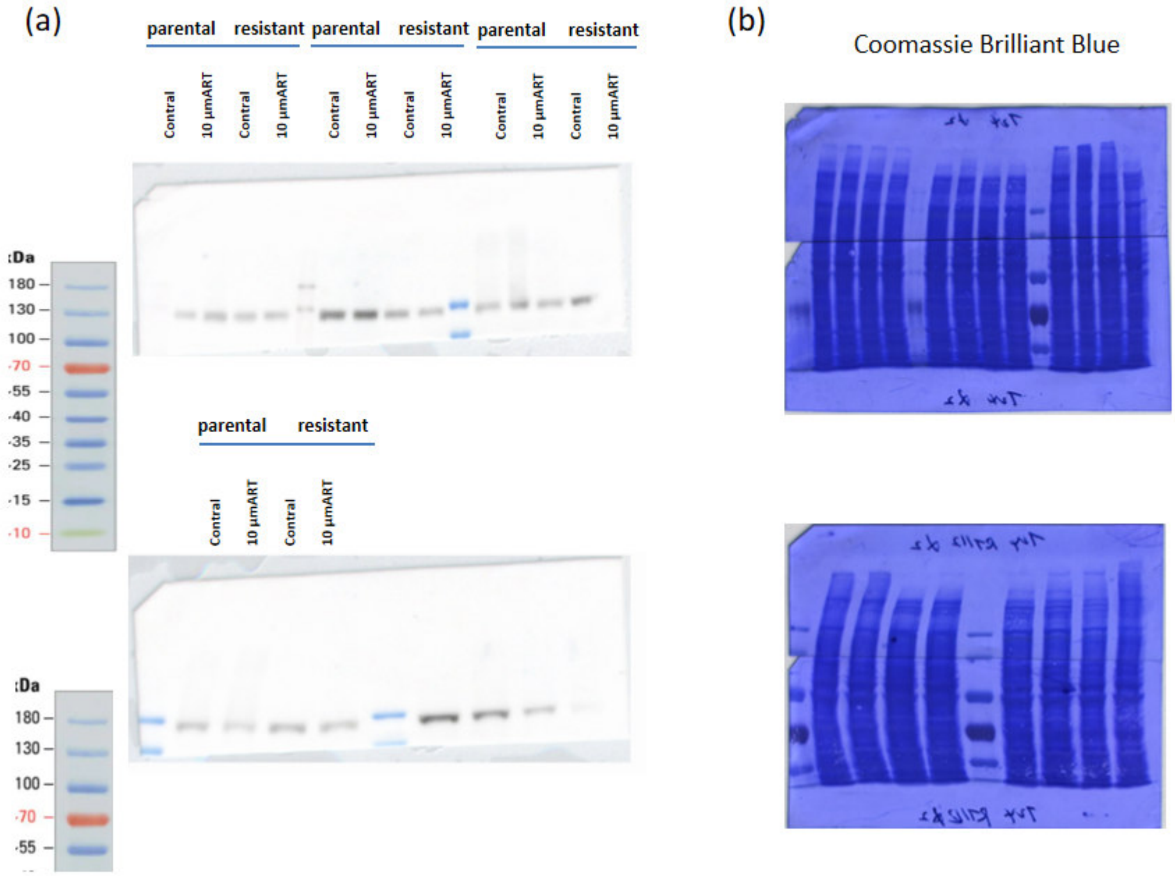

Figure S1.8: Detailed information about Figure 5 - Total protein expression of integrin proteins and integrin-related signaling proteins of T24 cells. (a) Protein expression of integrin α2 (150 kDa) and (b) corresponding Coomassie blue staining of total protein.

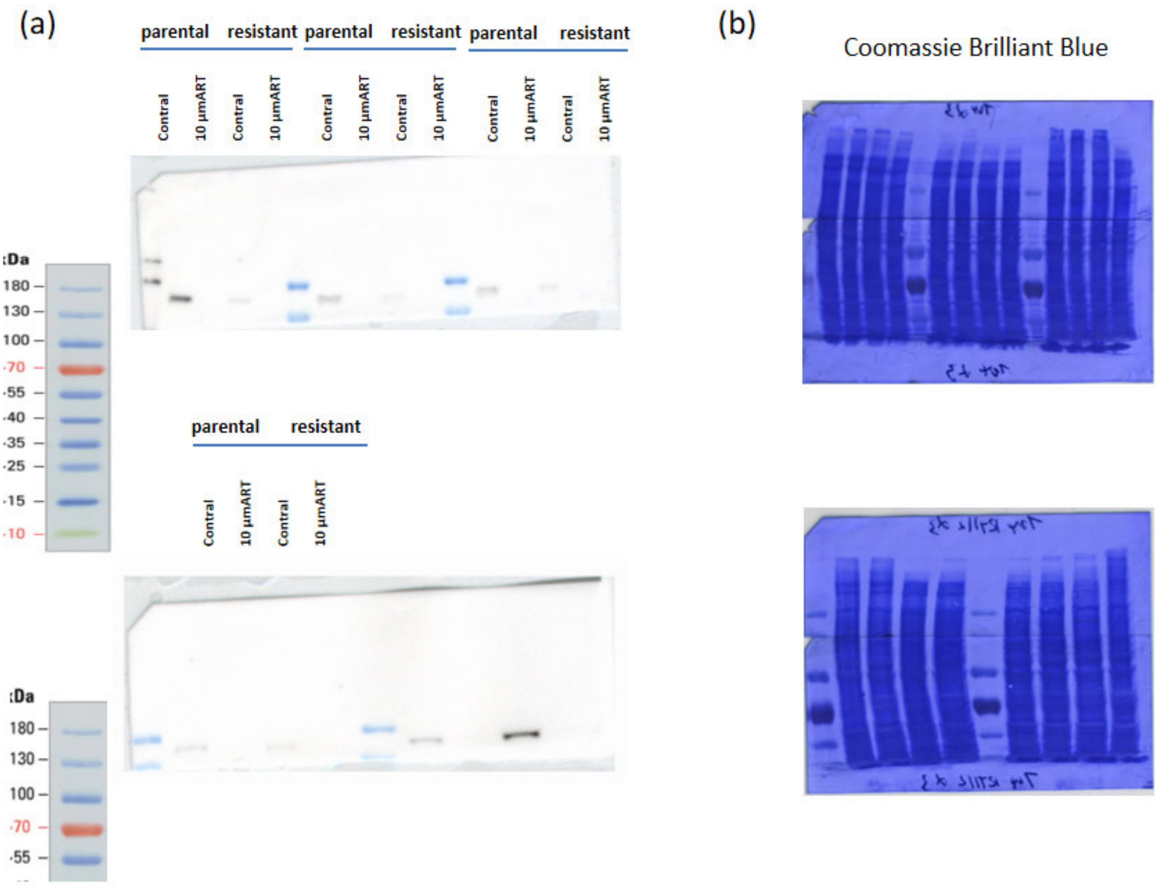

Figure S1.9: Detailed information about Figure 5 - Total protein expression of integrin proteins and integrin-related signaling proteins of T24 cells. (a) Protein expression of integrin α3 (150 kDa) and (b) corresponding Coomassie blue staining of total protein.

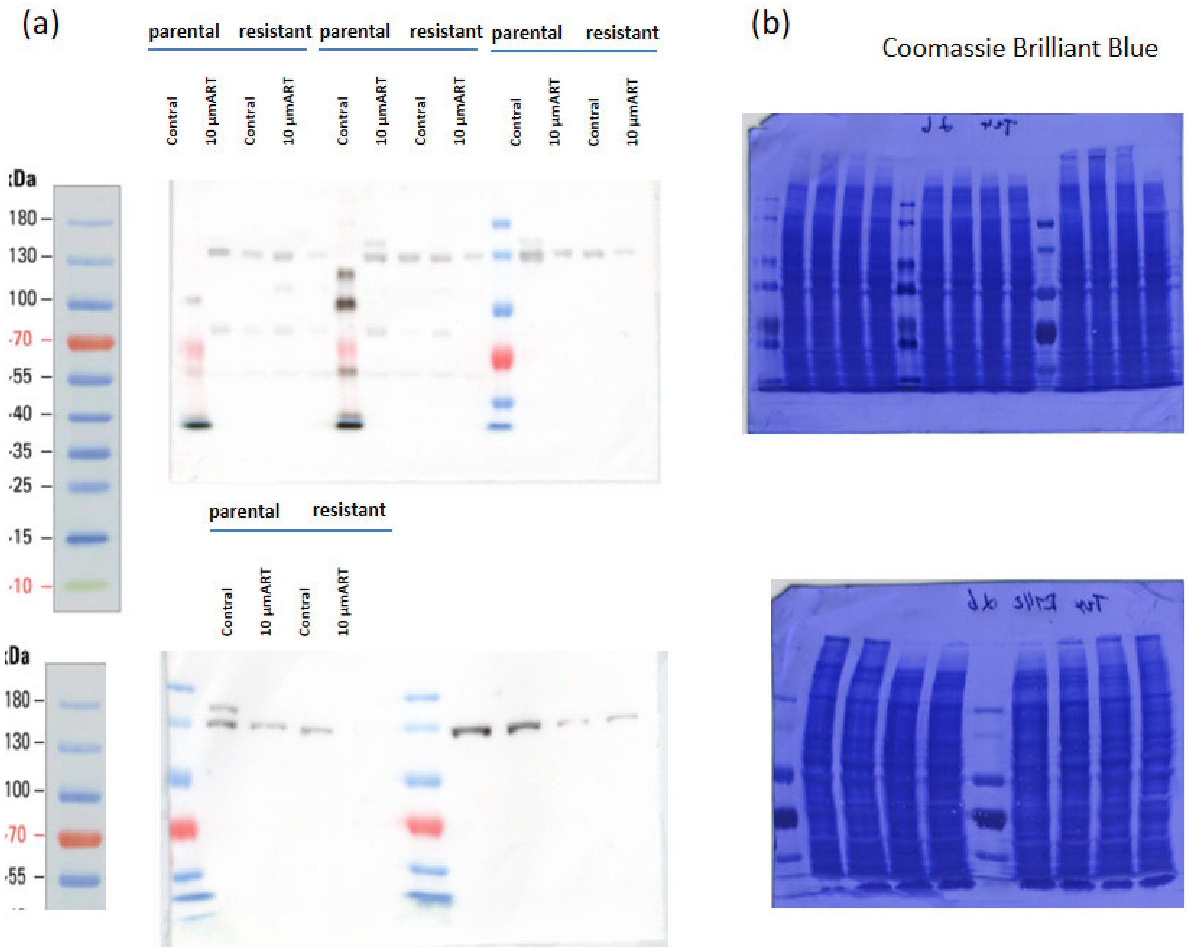

Figure S1.10: Detailed information about Figure 5 - Total protein expression of integrin proteins and integrin-related signaling proteins of T24 cells. (a) Protein expression of integrin α6 (150,125 KDa) and (b) corresponding Coomassie blue staining of total protein.

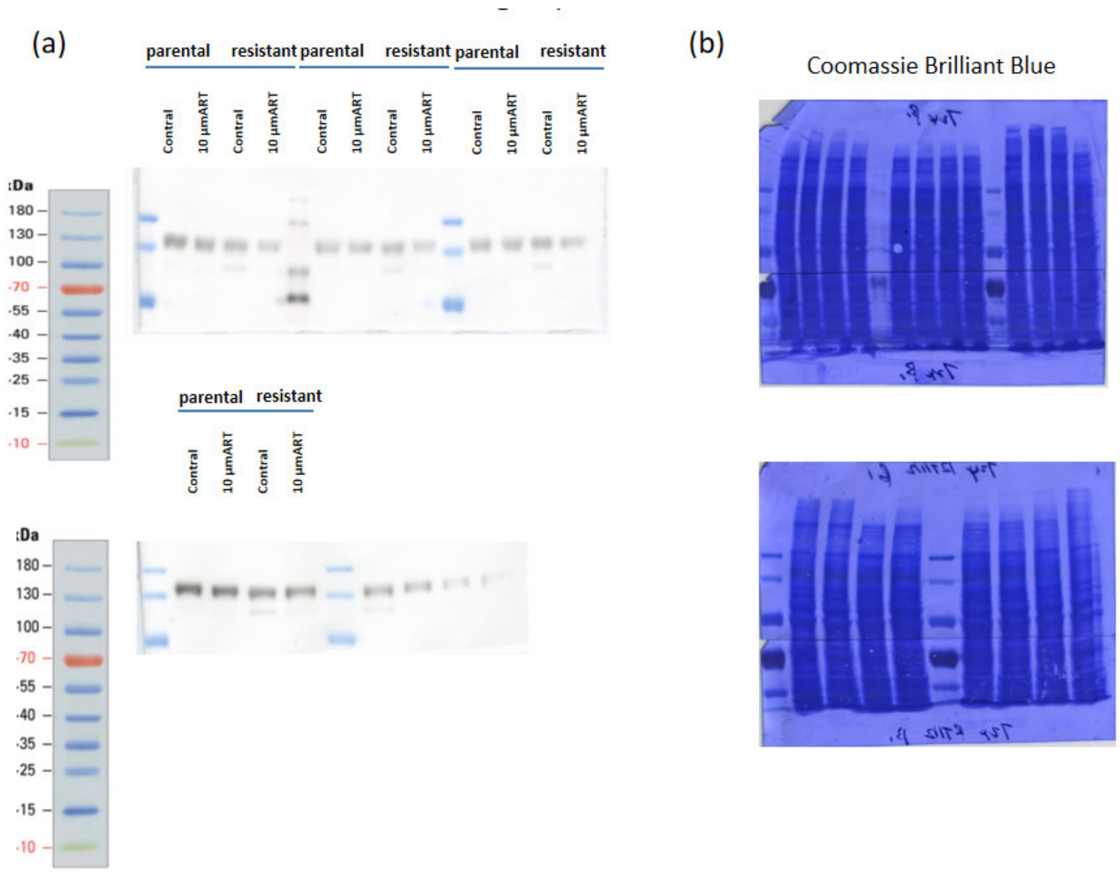

Figure S1.11: Detailed information about Figure 5 - Total protein expression of integrin proteins and integrin-related signaling proteins of T24 cells. (a) Protein expression of integrin β1 (130 kDa) and (b) corresponding Coomassie blue staining of total protein.

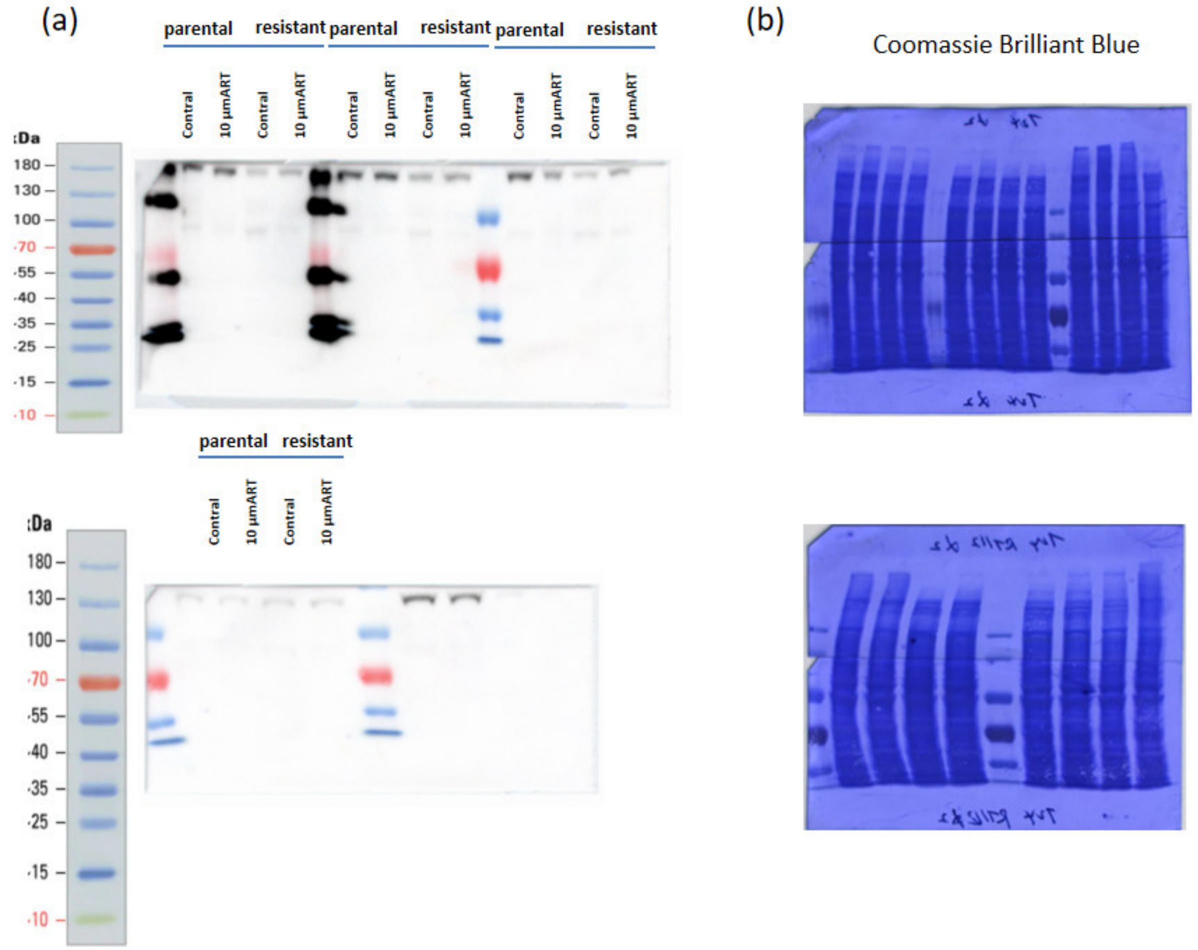

Figure S1.12: Detailed information about Figure 5 - Total protein expression of integrin proteins and integrin-related signaling proteins of T24 cells. (a) Protein expression of FAK (116-125 kDa) and (b) corresponding Coomassie blue staining of total protein.

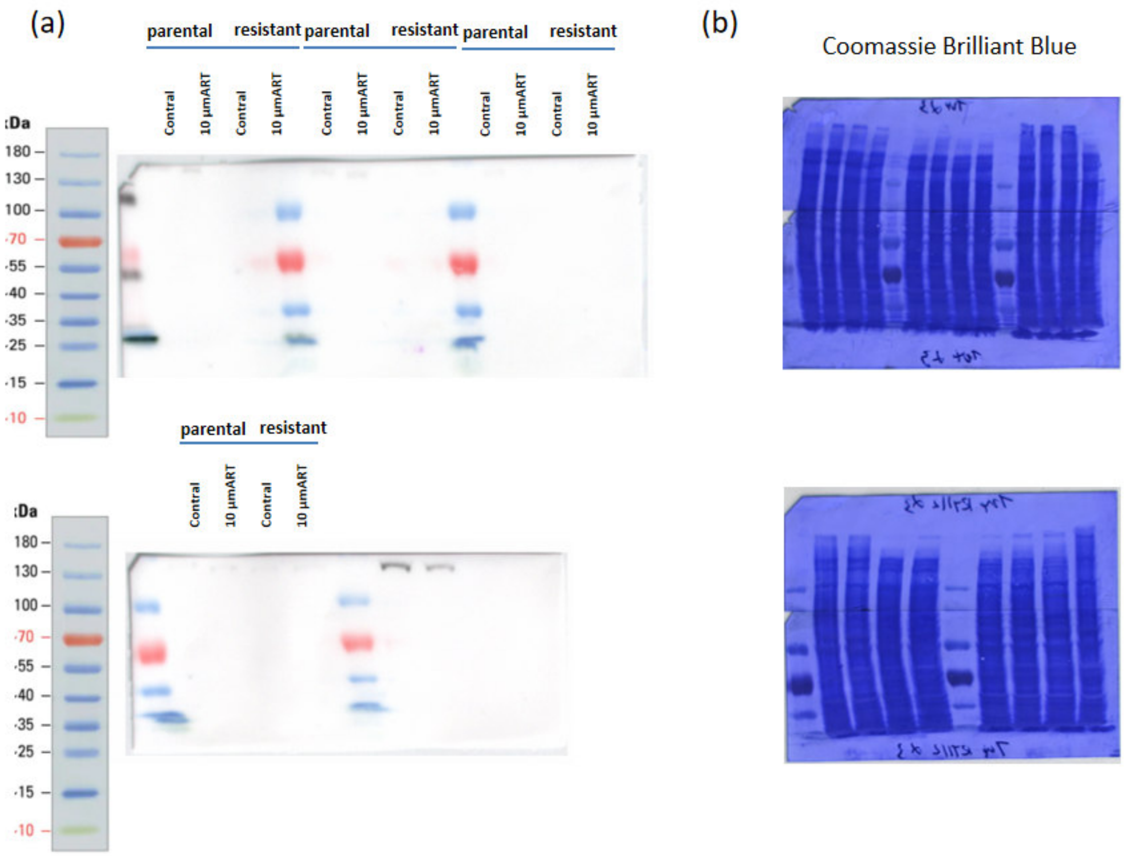

Figure S1.13: Detailed information about Figure 5 - Total protein expression of integrin proteins and integrin-related signaling proteins of T24 cells. (a) Protein expression of pFAK (116-125 kDa) and (b) corresponding Coomassie blue staining of total protein.

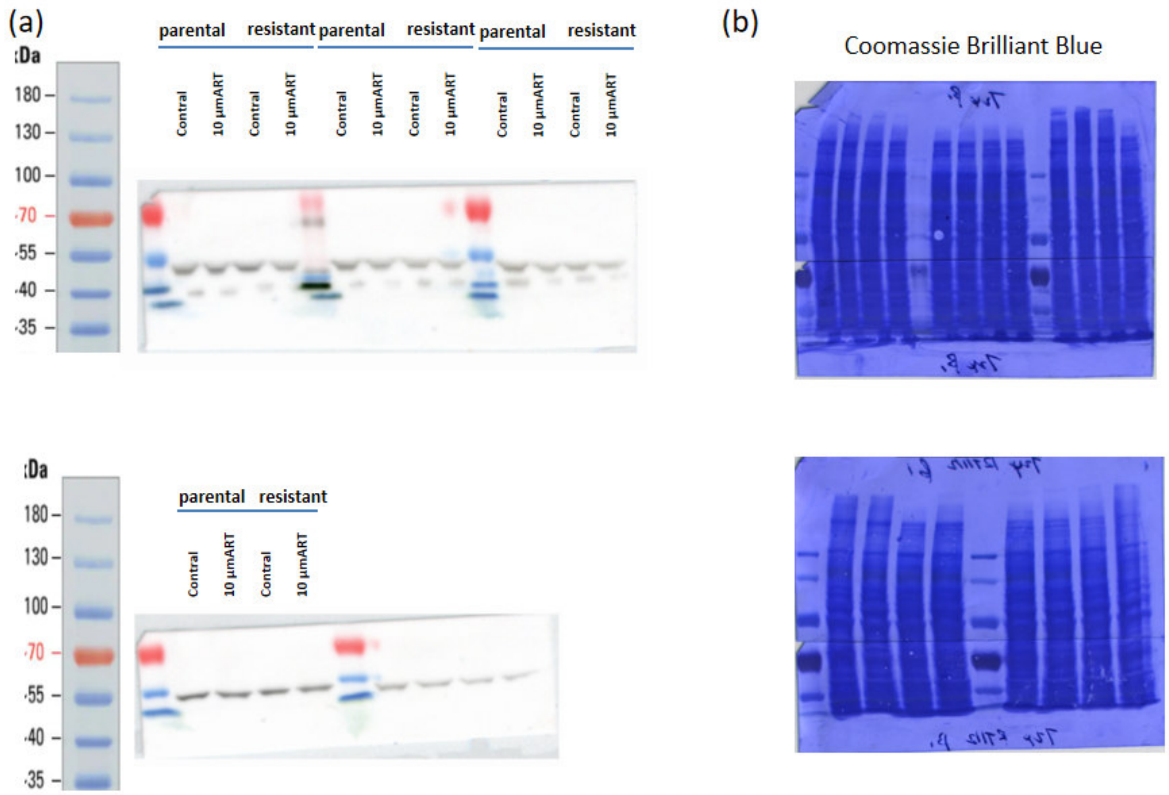

Figure S1.14: Detailed information about Figure 5 - Total protein expression of integrin proteins and integrin-related signaling proteins of T24 cells. (a) Protein expression of ILK (50 kDa) and (b) corresponding Coomassie blue staining of total protein.
